# An integrative view on sex differences in brain tumors

**DOI:** 10.1007/s00018-015-1930-2

**Published:** 2015-05-19

**Authors:** Tao Sun, Anya Plutynski, Stacey Ward, Joshua B. Rubin

**Affiliations:** 1 Department of Pediatrics, Washington University School of Medicine, St Louis, USA; 2 Department of Philosophy, Washington University in St Louis, St Louis, USA; 3 Department of Anatomy and Neurobiology, Washington University School of Medicine, 660 South Euclid Ave, St Louis, MO 63110 USA; 4 Campus Box 8208, 660 South Euclid Ave, St Louis, MO 63110 USA

**Keywords:** Metabolism, Cancer, Immunity, Sex determination, Evolution, Sexual dimorphism

## Abstract

Sex differences in human health and disease can range from undetectable to profound. Differences in brain tumor rates and outcome are evident in males and females throughout the world and regardless of age. These observations indicate that fundamental aspects of sex determination can impact the biology of brain tumors. It is likely that optimal personalized approaches to the treatment of male and female brain tumor patients will require recognizing and understanding the ways in which the biology of their tumors can differ. It is our view that sex-specific approaches to brain tumor screening and care will be enhanced by rigorously documenting differences in brain tumor rates and outcomes in males and females, and understanding the developmental and evolutionary origins of sex differences. Here we offer such an integrative perspective on brain tumors. It is our intent to encourage the consideration of sex differences in clinical and basic scientific investigations.

## Introduction

Sex differences in human development, aging, and disease are so commonplace they sometimes seem to escape questioning. Yet, the ways in which males and females consistently differ have significant importance for disease risk and prognoses, and cancer is no exception. There are measureable differences between men and women in cancer prevalence, mortality, and progression. Understanding these differences requires tracing their sources at the molecular, cellular, tissue, organismal and evolutionary levels, and providing an integrative explanation, one where data from different fields are mutually instructive and mutually constraining. This review aims at exactly this: to show how significant sex differences in brain cancers may be provided with an integrative explanation, drawing upon molecular, cellular, tissue, organismal, developmental, and evolutionary biology.

Beyond its inherent importance to normal biology, the study of sex differences can provide a novel perspective: a parallax view of the particular disease of interest. Moreover, it has the potential to uncover hidden elements of pathobiology. In order for sex to affect disease phenotype, it must affect fundamental mechanisms of risk and progression. Sex exerts its influence through differences in sex chromosome complement, epigenetic (organizational) and acute (activational) effects of sex hormone action, and sex-specific maintenance or reprogramming of maternal and paternal gene imprinting. As a consequence of these genetic and epigenetic processes, males and females exhibit different normal physiologies as well as different reactions to developmental, metabolic, and genotoxic stressors. These compensatory reactions often produce further sex-specific changes in epigenetics and gene expression that can widen the differences between males and females for disease risk.

Here, we will first examine sex differences in brain tumors. We will present data regarding the predominance of cases in males for most individual tumor types and consider the lessons of exceptions like meningioma and low-grade astrocytoma. Second, we will review the developmental and evolutionary basis for sex differences. It is only through recognizing how evolution has favored different growth optima in males and females and how the effects that sex determination through development have on gene expression, metabolism, growth, immune function, and homeostatic responses to stressors that we can hope to recognize and anticipate the effects of sex in cancer diagnosis and therapeutic responses. We will conclude by contemplating how incorporating the biology of sex differences can advance neuro-oncology care and research.

## Sex differences in brain tumor incidence and outcome

### Primary brain tumors

With few exceptions, sex differences are evident in tumor incidence and mortality throughout the world and regardless of race or age (http://globocan.iarc.fr/Pages/fact_sheets_cancer.aspx). Cancers that affect both males and females in all tissues and organ systems frequently exhibit male:female incidence ratios that range from approximately 1.5:1–3:1 (American Cancer Society (http://www.cancer.org/acs/groups/content/@editorial/documents/document/acspc-044552.pdf) and the Cancer Research UK (http://www.cancerresearchuk.org/cancer-info/cancerstats/incidence/). Males not only develop more cancers, they frequently have poorer responses to therapy as measured by event-free and overall survival [1–10]. Therefore, a closer examination of sex differences in cancer is likely to reveal differences in fundamental mechanisms of tumor initiation, tumor promotion, and therapeutic response. Central to this endeavor will be delineating distinct relationships between sex differences and specific oncogenic mechanisms or molecular subtypes of cancer, as well as between sex differences and putative cells of origins for given tumor types. Here we will consider four different brain tumor types to illustrate how patterns of sex differences might be used to identify potential molecular and cellular mechanisms through which sex affects cancer biology.

In adults, gliomas account for the vast majority of primary parenchymal brain tumors. This heterogeneous group of neoplasms includes those with features of astrocytes, oligodendrocytes, and ependymal cells, and ranges from World Health Organization (WHO) grade I to IV disease. With few exceptions, gliomas occur more commonly in males and do so with some restriction to specific oncogenic mechanisms [11]. Four molecular subtypes of glioblastoma (GBM) are recognized [12, 13]. The *Mesenchymal* subtype, which is characterized by combined loss of neurofibromin (NF1), PTEN, and TP53 function, exhibits the greatest and most consistent disparity in male to female rates (2:1) [14]. The *Proneural* subtype (*TP53* mutation, isocitrate dehydrogenase (*IDH*) mutation, platelet-derived growth factor receptor (*PDGFRA*) amplification) and *Neural* subtype (epidermal growth factor receptor (*EGFR*) amplification, *TP53* mutation, *CDKN2A* deletion) also exhibit sex differences that approximate those of *Mesenchymal* subtype tumors. In contrast, the *Classical* subtype (*EGFR* mutation/amplification, *CDKN2A* deletion) occurs equally in males and females. Further distinctions between those oncogenic events that exhibit differential modulation by sex will be critical to understanding how sex affects cancer incidence and outcome.

Disparity in the rates of brain tumor formation in prepubertal boys and girls is particularly instructive with regard to possible mechanisms by which sex differences affect cancer. The peak incidence of pediatric brain tumors occurs in children less than 4 years of age, when circulating sex hormones are at a lifetime nadir [15, 16]. Similar to what is observed in adult GBM, sex appears to exhibit molecular subtype-specific effects in pediatric brain cancers. This is well illustrated by medulloblastoma, ependymoma, and pediatric high-grade glioma. Medulloblastoma, the most common malignant brain tumor of childhood, comprises four distinct subtypes based on gene expression, mutation, and copy number variations [17–20]. The four subtypes are known as the *Wnt*, the *Sonic Hedgehog* and *Group 3* and *Group 4*. The different subtypes have been hypothesized to originate from discrete neural progenitor populations that are present during normal cerebellar development [21–26].

Only *Group 3* and *Group 4* medulloblastomas exhibit a 2:1 male to female incidence ratio. Both subtypes possess isochromosome 17q (loss of chromosome 17p with a duplication of 17q), and *Group 3* tumors also exhibit amplification of CMYC. Interestingly, among female cases of Group 3 medulloblastoma, there is frequent loss of an X chromosome. *Groups 3* and *4* medulloblastoma also share chromatin abnormalities as a consequence of mutations in KDM6A and EZH2, which result in dysregulation of histone H3 lysine 27 (H3K27) trimethylation [27]. In contrast, the other two molecular subtypes of medulloblastoma do not possess isochromosome 17q exhibit mutational activation of the Wnt or Sonic hedgehog pathways and genetic mutations that affect NCor and SWI/SNF chromatin remodeling complexes [27]. These subtypes occur with equal sex distribution.

Similar to medulloblastoma, pediatric ependymoma also occurs with an overall male:female incidence ratio of 2:1 as a result of a sex differences within specific subtypes of disease. In a multi-institutional study by Witt et al. [28], ependymomas from two separate cohorts of patients were stratified into three groups: supratentorial, posterior fossa, and spinal and posterior fossa, by consensus hierarchical clustering of transcriptional profiles. The posterior fossa (PF) ependymomas were further subdivided into Group A and Group B tumors. Group A PF ependymoma occurred more commonly in males with a median age of presentation of 2.5 years, while Group B PF ependymoma occurred equally among older males and females (median age of presentation was 20 years). In addition to sex and age of presentation differences, Group A and Group B PF ependymoma also diverge in their apparent oncogenic mechanisms. Group A PF ependymomas exhibit a methylator phenotype suggesting epigenetic origins of dysregulation in multiple cancer-related pathways [28, 29]. Group B PF tumors did not possess a methylator signature and instead were distinguished by alteration in ciliogenesis and metabolism [28]. Like medulloblastoma, the different subtypes of ependymoma may derive from different cells of origin [30, 31].

Epigenetic mechanisms of tumorigenesis are also likely in pediatric high-grade gliomas where histone mutations are frequent [32–39]. In deep midline GBM of early childhood, lysine 27 is often mutated to methionine (K27M) in histones H3.1 or H3.3. This results in global changes in H3K27 trimethylation. Importantly, these GBM also exhibit a male:female incidence of approximately 2:1, while those GBM that occur in young children without histone mutation and by other oncogenic mechanisms such as RB1 mutation [33] do not.

Taken together, the sex effects in medulloblastoma, ependymoma, and pediatric high-grade glioma suggest that specific mechanisms of tumorigenesis and particular populations of progenitor cells may be most sensitive to the influence of sex. Both ependymoma and high-grade glioma exhibit the strongest effects of sex in subgroups in which epigenetic mechanisms appear to drive tumorigenesis. These observations raise the question of whether epigenetic mechanisms of brain tumorigenesis may be particularly sensitive to the effects of sex. Further, both medulloblastoma and pediatric high-grade glioma exhibit sex effects in subsets of disease in which there is dysregulation of histone H3K27 trimethylation, potentially providing reason to focus on this particular aspect of epigenetics. As discussed below, early sex determination is a period of large-scale epigenetic change as a consequence of the organizing effects of sex hormones. Finally, in all three tumors, sex differences are limited to specific subtypes, which could reflect sex-specific vulnerabilities within particular progenitor populations.

Molecular subtype-specific sex effects can also be observed in brain tumors that do not exhibit sex differences in incidence. While supratentorial ependymoma occurs with equal overall frequency in males and females, the Gilbertson group recently reported a novel fusion oncogene in supratentorial ependymoma with a significant male prevalence (M/F = 2/1) [40]. Similarly, Warrington et al. [41] recently reported sex-specific effects of polymorphisms in adenylate cyclase 8 in individuals with NF1 and glioma, despite an equal incidence of low-grade glioma in males and females. These studies indicate that further subgroups exist within these diseases and raise the possibility that male and female brain tumor patients may require different approaches to treatment under certain circumstances.

### Secondary brain tumors

Secondary brain tumors or brain metastases can complicate many cancers, especially those primary diseases involving the lung, breast, colon, kidney, or skin [42]. The incidence of secondary brain tumors appears to be on the rise. A prospective study by Smedby et al. [43] showed the rate has doubled in Sweden over the past two decades. This dramatic change in brain metastases is largely attributed to advancements in primary cancer care, including better early detection and frontline therapies which lead to longer initial survival and a greater rate of death from recurrent and disseminated disease [44]. Excluding sex-specific cancers like breast cancer, males have significantly higher rates of brain metastases than females in almost all cancer types [11, 45].

Brain metastasis is a complicated process involving epithelial-mesenchymal transition (EMT), evasion of immune surveillance, crossing the blood–brain barrier, and establishment of tumor growth within the brain parenchyma [46]. During each step, there may be sex-specific biological differences that contribute to the overall sex disparity in brain metastasis rates. There have been few direct analyses of possible sex differences in EMT. In a lung cancer model, it was observed that periostin expression, a marker for EMT, was more highly expressed in biopsies from male patients compared to those from female patients [47]. Periostin has been demonstrated to enhance glioblastoma stem cell function [48]. As detailed below, immune surveillance is profoundly different in males and females, and a recent study demonstrates that there are sex differences in blood–brain barrier permeability in multiple sclerosis [49].

### Meningioma

Meningiomas occur with a striking sex disparity. They are more than twice as likely to occur in adult females, ages 30–70, and risk factors include radiation exposure, increasing age, and genetic predisposition syndromes such as Neurofibromatosis type 2 (mutation in the *Merlin* gene) and Gorlin syndrome (mutation in the *Patched* gene) [50]. In addition to the differences in their incidence, male and female meningiomas differ in their grade: meningiomas in females are more commonly low grade, while those in males are more commonly malignant.

A growing body of evidence links meningiomas to acute (activational) effects of sex steroids. First, meningiomas rarely develop in prepubertal children when circulating sex hormones are low. Second, women develop meningiomas 2½ times more frequently than men; and third, meningiomas are known to express progesterone and estrogen receptors [51, 52]. In fact, hormone replacement therapy in women has been shown to increase the risk of meningioma [53]. In addition, there are case reports of rapid growth of meningiomas during pregnancy, which then stop growing after delivery [54–58]. Meningiomas have not been linked to female oral contraceptive use, suggesting that either higher or normal variation in estrogen and progesterone action may be required for tumorigenic effects [59, 60].

In addition to potential tumor-promoting effects of estrogen and/or progesterone, disparities in meningioma rates may also be the product of protective effects of testosterone. In men with prostate cancer undergoing androgen deprivation therapy, there is an increased risk of meningioma growth. Meningioma biopsies express luteinizing hormone-releasing hormone receptor, and it is thought that high testosterone levels may be protective against meningioma [61, 62]. In addition, there are four case reports in the literature detailing meningioma formation and growth in male-to-female transsexual patients undergoing cross-sex hormone therapy [63–66]. The administration of estradiol and androgen antagonist cyprotenone acetate resulted in the development of meningiomas requiring treatment. Discontinuation of cyprotenone acetate in one patient was correlated with tumor regression [66]. The protective effects of androgens are also suggested by increased meningioma incidence in females treated with cyprotenone acetate. It is possible that the apparent exception of meningioma to the pattern of male predominance in intracranial tumors is related to its strong link to sex hormone action.

## Mechanisms of sex effects in cancer

**Fig. 1:**
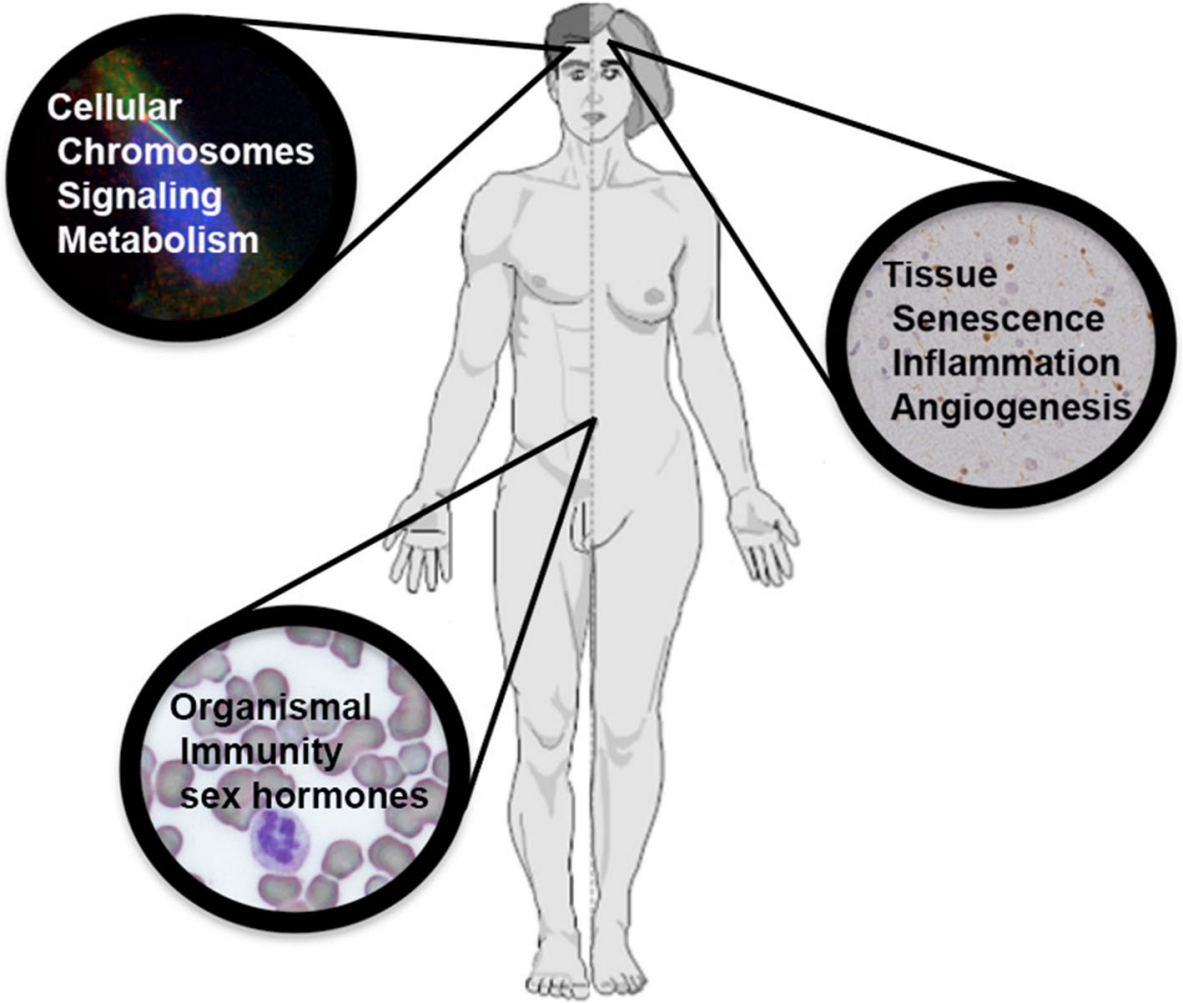
Mechanisms underlying sex differences in cancer: effects of sex on cancer occur at the level of cellular transformation, tumor tissue organization, and organismal processes that impact on cancer occurrence and outcome. Pictured are processes discussed in the text. Background to the cellular processes is a medulloblastoma cell with the nucleus in *blue* and a primary cilium evident in green overlying the nucleus. Background to the tissue processes is CXCL12 expression (*brown*) in glioma tissue. Background to the organismal processes is a blood smear with a neutrophil

The mechanisms by which sex can affect cancer rates and outcome range from the cellular to the organismal level. For examples, cellular and organismal metabolism and growth rates differ as a function of sex. Immune function is profoundly different in males and females, and the disparity in the incidence of cardiovascular disease suggests vessel function may be fundamentally different. Here we will review cell intrinsic and non-cell intrinsic mechanisms by which sex can exert an effect on cancer (Fig. 1).

### Cell intrinsic mechanisms

#### P53

At the core of human cancer cell biology is the inactivation of RB1 and p53 function [67]. Each of these tumor suppressors plays an indispensible role in maintaining normal cell number and preservation of genomic stability and function. Changes in p53 function can have different consequences in males and females in multiple species. The first description of sexual dimorphism in the p53 pathway was in mice with complete loss of p53 function. Initial characterization of these mice suggested that they underwent normal development but had increased rates of spontaneous tumor formation [68]. Closer examination of neural development revealed that nearly 25 % of female *p53−*/*−* embryos developed neural tube defects (NTD) and died [69]. The sex-specific effect of p53 loss on neural tube closure has been ascribed to differences in X gene dosage [70]. In addition, the zinc finger protein, Lyar, has been shown to function synergistically with p53 loss in promoting NTDs and death of female embryos [71].

Given the importance of p53 function for tumor suppression, it is not surprising that sexual dimorphism in the p53 pathway could impact on cellular transformation. We recently demonstrated that upon loss of p53 function, male and female *Nf1−*/*−* astrocytes exhibited significantly different growth rates and clonogenic potential. Male *Nf1−*/*−*; *DNp53* astrocytes grew four times faster than female *Nf1−*/*−*; *DNp53* astrocytes and exhibited an approximately ten-fold increase in clonogenic cell frequency [14]. These sex differences in loss of p53 function contributed to differences in transformation and in vivo tumorigenesis.

Although overexpression of p53 ordinarily leads to embryonic lethality in mice, a mouse model with a single allele of p53 hyperactivation exhibited reduced incidence of spontaneous tumors [72]. Unexpectedly, gain of p53 function was also associated with signs of premature aging. In drosophila, male and female flies exhibited different responses to stress-induced p53 activation, and upregulation of p53 promoted a longer life in male flies, while reducing lifespan in females [73]. Sex differences in human life span are evident across the globe, and females are well known to live longer lives than males. Since cancer risk rises with age, sexual dimorphism in the p53 pathway may have differential impacts on human aging and cancer risk secondary to it.

#### RB1

We recently demonstrated that significant sex differences exist in the regulation of RB1 function in mice. Using a murine model of glioblastoma in which astrocytes served as the cell of origin, we showed loss of p53 function resulted in a remarkable increase in in vitro growth as well as in vivo tumorigenicity in male, but not female, astrocytes [14]. We further demonstrated this is due to sexually dimorphic RB1 activity in the astrocytes, and when both RB1 and p53 functions were depleted, both male and female astrocytes showed a similar potential for forming tumors. This evidence indicates that sexual dimorphism exists upstream of RB1 function and that when RB1 is intact, sex differences in its regulation can have significant effects on malignant transformation. Consistent with our findings, adult and pediatric high-grade gliomas with RB1 mutation occur with equal frequency in males and females ([33] and our unpublished TCGA data analysis). Moreover, RB1 and CDKN2A loss are mutually exclusive genetic events in GBM, suggesting equivalent consequences of their loss of function (our unpublished TCGA data analysis). CDKN2A loss is a signature event in *Classical* GBM, which also occurs with equal frequency in males and females [14].

Similar to p53, Rb functions as a node for integrating signals regarding genomic stability, proliferation, apoptosis, stem cell function, and differentiation. Our recent findings may have significant implications in both basic research of cancer biology and clinical practice. For example, ongoing clinical trials are evaluating the use of CDK inhibitors in the treatment of RB1 intact cancers. CDKs are upstream regulators of RB1 function, and thus it will be important to determine whether there are sex differences in response.

#### cAMP

Sex differences are also evident in other cell signaling pathways. We recently demonstrated that both cAMP synthesis and degradation differ in male and female astrocytes, resulting in differences in basal cAMP levels [41]. A relationship between intracellular cAMP levels and human cancers was established decades ago [74]. A similar relationship is evident in a number of important mouse models in which low cAMP levels favor tumor cell growth and cAMP-elevating drugs like Rolipram exhibit potent anti-tumor activity [75–78]. Among these models is one of Neurofibromatosis type 1 (NF1)-associated optic pathway glioma [79]. In this model, we were able to alter the stereotypical pattern of glioma formation through forced expression of the cAMP-specific phosphodiesterase PDE4A1 and focal reductions in cAMP levels [75]. These data indicated that cAMP levels were a determinant of tumorigenesis in NF1. Based on these results, we performed a human genome-wide association study and found that polymorphisms in the adenylate cyclase 8 gene were correlated with glioma risk in NF1 but in a sex-specific fashion, elevating risk in females and suppressing risk in males [41]. These data indicate that sex might play a role in the overall contribution of cAMP to gliomagenesis of NF1 and possibly in other brain tumors. It also raises the question of whether male and female patients would respond differently to the cAMP-elevating drugs.

### Non-cell intrinsic mechanisms

#### Sex differences in immune function

The immune system plays an essential role in cancer prevention, and harnessing its power is a goal of a number of current experimental treatments. However, profound differences exist in immune function between males and females, and these may need to be addressed in treatment protocols. Differences in male and female immune function are most evident in autoimmune diseases. Multiple sclerosis (MS) and rheumatoid arthritis (RA) occur twice as commonly in females compared to males [80, 81] and greater than 90 % of all Systemic Lupus Erythematosus cases are in females [46, 82].

The mechanism(s) underlying these sex differences are likely to be complex, but include sex hormones acting through their classic steroid receptors (i.e., estrogen and androgen receptors) as well as non-canonical receptors (i.e., GPR30) expressed by immune effector cells [83]. Estrodial (E2), acting through its receptor ER alpha, promotes expansion of antigen-specific CD4 T cells and Th1 cells. These in turn produce interferon—gamma [84]. E2 has also been demonstrated to enhance interferon—gamma production by natural killer (NK) cells [85]. In contrast, androgens directly inhibit T-helper cell differentiation and suppress the adaptive immune response [86]. In addition to sex hormone actions, X chromosome encoded alleles, including several microRNAs, are known to exert direct effects on immune function [87, 88].

While sex differences in lymphocyte function may contribute to anti-brain tumor immune activity, it is the microglia that are the primary effectors of central nervous system immunity [89]. Long-lived resident microglia populate the neural tube early in development [90]. They derive from the macrophage–monocyte lineage, and through their phagocytic functions and their elaboration of cytokines, growth factors and chemokines, they play critical roles in regulating neural stem cell function, cell number, and synaptogenesis during normal development [89, 91–93].

Resident microglia as well as infiltrating bone marrow-derived macrophages/microglia are active in pathological conditions including brain tumors. Microglia are abundant in human glioma [94] but their precise roles remain unclear. In some cases, pro-tumorigenic roles for microglia have been observed, such as expansion and migration of glioma cells in response to microglia-derived signals [95–97]. In contrast, other studies showed that the activation of microglia and macrophages decreased tumor growth in mice, indicating that tumor-associated microglia and macrophages may have anti-tumor effects [98–100]. In line with these results, genetic depletion of macrophages increased glioma growth in vivo [101]. Significant sex differences exist in the numbers and activity of microglia throughout life [88, 102–104]. It will be interesting to determine whether any of the variability in apparent tumor-associated roles for microglia is sex dependent.

Astrocytes are also important effectors of central nervous system immune response. Astrocytes have well-established sexually dimorphic behavior, particularly in the elaboration of cytokines. Inflammation-associated astrocyte production of IL-1β, IL-6, and TNF-α is greater in male compared to female astrocytes [105]. Each of these cytokines can stimulate the growth of experimental cancers and may contribute to sex differences in brain tumor rates.

#### Sex differences of aging stroma

Context matters: whether a transformed cell eventuates into a tumor has a great deal to do with the surrounding tissue microenvironment. Healthy stroma can prevent the progression of cancer, and tissue disruption can induce progression. Striking examples of the role of stroma in tumor formation include the induction of mammary carcinomas in mice after implantation of normal epithelial cells into mutagenized mammary fat pads but not when mutagenized epithelial cells were implanted into control fat pads [106], the production of tumors in RSV expressing chickens only in the setting of inflammation [107], and the injection of teratocarcinoma cancer into a mouse blastocyst leading to healthy normal tissues [108].

Tissue-based cancer-suppressive mechanisms may underlie the relegation of most cancers to old age. Aging stroma is more permissive for cancer progression, and the properties of aging stroma are in turn often governed by sex differences. There are both direct and indirect links between sex, the aging stroma, and cancer. One of the most profound sex differences in aging stroma exists in the differential inflammatory response, as discussed above. There is an innate, bidirectional interaction between inflammatory mediators and sex steroid hormones [109]. Females are less susceptible to hepatocellular carcinoma—the most common cancer of the liver—which is often caused by chronic inflammation due to hepatitis viral infection. Colorectal cancer is also more common in men than women, and the difference is more striking among premenopausal women and age-matched men.

Another potentially important difference in aging stroma may be linked to telomere shortening in men [110]. While it is unclear whether telomere shortening is a causative or correlative factor in mortality [111], it is uncontroversial that on average, men have shorter telomeres than women. Telomere down-regulation is common as we age, and the rate at which protective telomere chromosome caps are lost is higher in men. Because critically short telomeres induce cellular replicative senescence or cell death, by removing telomerase-driven maintenance, telomere shortening limits the number of times a cell may divide, and so telomerase down-regulation may have evolved in response to the increased risk of cell immortalization and cancer that accompany larger size. So, size dimorphism may be a cause of increased telomerase shortening in men. Large size means more cell divisions as we age, and thus potentially a greater risk of cell immortalization and cancer risk. However, estrogen directly activates a promoter of telomerase, and so may also indirectly affect differential rates of shortening as we age [112].

Sex chromosome haploidy may also differentially affect aging stroma and thus lifespan; this has been called the “unprotected X chromosome” theory. Males are the heterogametic sex and are vulnerable to a variety of X-linked diseases, where only a single loss of function mutation is needed for diseases such as hemophilia, Lesch Nyhan syndrome, and muscular dystrophy. Females are mosaics, with stochastic inactivation in each cell of one or the other X chromosome; this mosaicism is arguably protective against disease. As females develop, there is a skew in the ratio of X-inactivation, presumably due to the greater fitness of one or another chromosome.

Cumulative damage to cells due to oxidative stress may also differentially affect the sexes, in part due to sex differences in size, or possibly due to hormone-mediated production of ROS (reactive oxygen species). Estrogen reduces the production of ROS and is a potent antioxidant and regulator of antioxidant genes; conversely, testosterone has no antioxidant properties [113, 114]. This has been called the “oxidative handicap hypothesis,” or the “mother’s curse,” given the role of mitochondrial DNA in oxidation. According to Tower [115], “mitochondrial malfunction should contribute to the aging phenotype, and perhaps do so in males more than females,” on the theory that mutations that are substantially harmful to males can accumulate in mitochondrial genomes if they are only slightly deleterious, neutral, or beneficial to females [116–120]. There is some experimental evidence that variance in naturally occurring mitochondrial haplotypes in *Drosophila melanogaster* affected lifespan and mortality rates in males but not in females, which is consistent with the idea of mutation load in mitochondrial genome affecting male aging [116]. However, a recent review argues that the experimental evidence is tenuous with respect to this hypothesis [121].

These differences in aging stroma are likely affected by a variety of proximate and more remote causal processes. As discussed below, there may be an evolutionary explanation. Differential sex-specific fitness—e.g., with respect to selection for various traits that affect fertility—could lead to antagonistic coevolution of traits that affect both sexually selected traits and cancer risk. Sexual selection could also lead to differential epigenetic effects. Parental imprinting can be sex specific, and may play a critical role in development and aging. Whether directly or indirectly, sex differences can lead to differences in how diet and environmental compounds affect cancer risk in males versus females.

#### Vascular function

An additional significant and potentially cancer relevant sex difference is the effects of sex on vascular function. Cardiovascular disease occurs with greater frequency in men and post-menopausal women than in premenopausal women [122]. The mechanistic basis for these well-established observations remains unclear and may include sex differences in response to early environmental stressors as well as acute vascular actions of sex hormones [123]. Potential hormone-dependent mechanisms include estrogen-stimulated vasodilatory effects of nitric oxide and prostacyclins, as well as anti-inflammatory and antioxidant effects of estrogen [124]. Given these demonstrated effects of estrogen, it is unclear why estrogen replacement therapy for post-menopausal women has been associated with adverse vascular events [125], and estrogen therapy for male to female transsexuals has been causally related to their increased cardiovascular disease [126]. Further, testosterone treatment of female to male transsexuals is not correlated with a change in cardiovascular disease frequency, and testosterone antagonist therapy in men with prostate cancer increases cardiovascular adverse events. Thus, there appear to be cardiovascular protective effects of testosterone [127]. While these observations indicate an incomplete understanding of the mechanisms by which sex affects cardiovascular function, they should stimulate consideration of whether sex differences in vascular function might extend to sex differences in tumor-associated angiogenesis as well as sex differences in endothelial cell effects on brain tumor stem cells. The perivascular space is an essential brain tumor stem cell niche. We, and others, have demonstrated that endothelial cell–glioblastoma stem cell crosstalk plays an essential role in inducing and maintaining the glioblastoma stem cell state [128]. This is critically important as perivascular niche function stimulates tumor growth, resistance to therapy and recurrence after treatment, and is known to involve nitric oxide metabolism [129].

## Developmental origins of sex differences

**Fig. 2:**
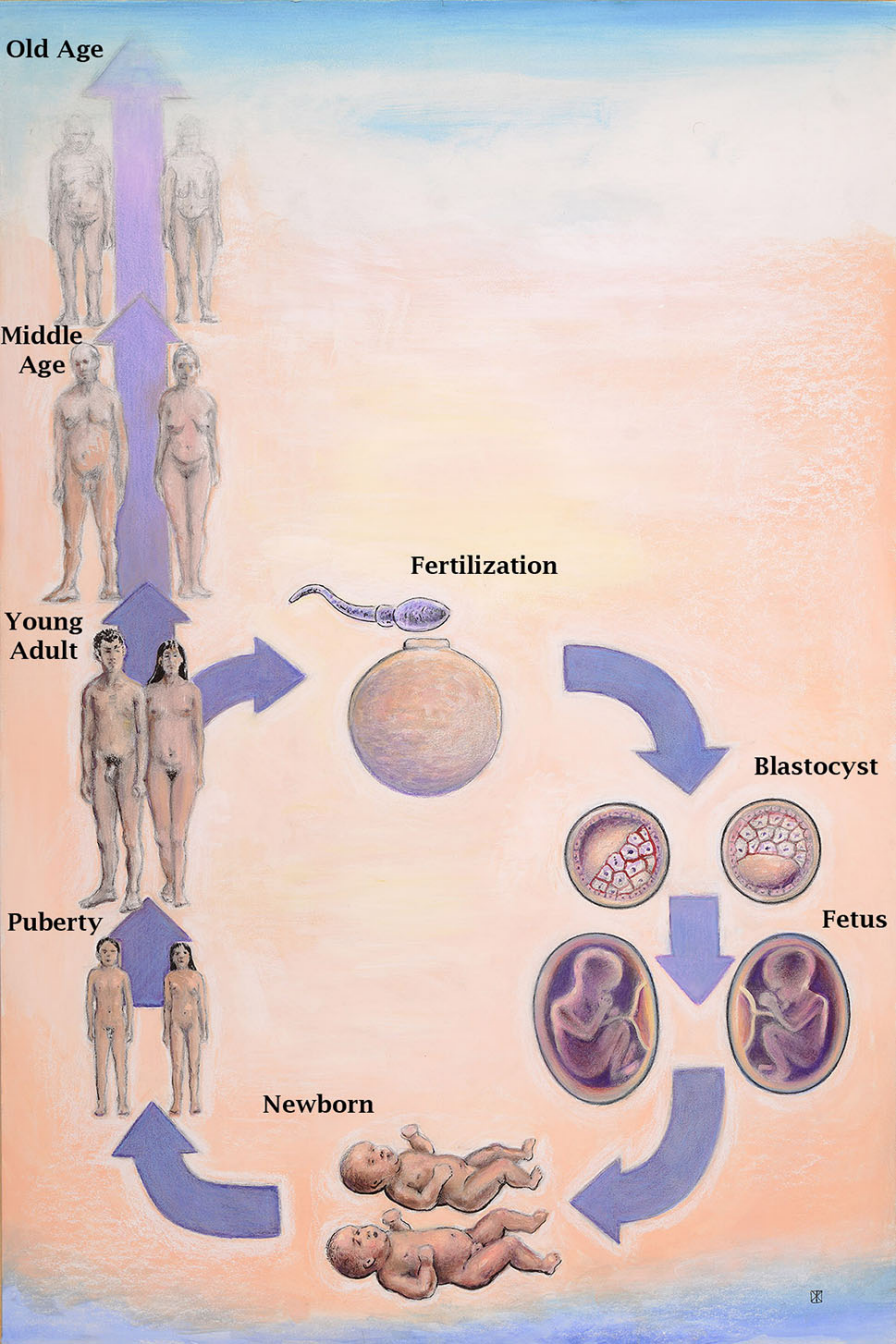
Developmental origins of sex differences: At all stages of life from fertilization until death, sex differences arise through direct effects of differences in sex chromosome complement, and the organizational and activational effects of sex hormones. Pictured are stages of development discussed in the text

The state of human health and disease is determined throughout life by genetics, epigenetics, environmental exposures, and aging-associated degradation of genomic, cellular, and tissue function. At each stage, from conception to death, males and females differ as a consequence of their chromosome complement, epigenetics, and hormone actions. Each of these component mechanisms has been shaped by evolution to create and maintain phenotypic differences between the sexes, a process collectively referred to as sexual determination [130]. While overt sex differences that emerge at each stage of normal development can range from subtle to profound, additional and significant underlying sex differences have been revealed under suboptimal growth conditions or frank pathological states. Differences in adaptations to in utero stressors may have particular relevance to this discussion in light of the ever-expanding body of data to support Developmental Origins of Health and Disease [131]. How normal sex determination and sexually differentiated function affects cancer risk is not understood. It is important to consider, however, as cancer occurs more commonly in males, and therefore sex differences in genetics, epigenetics, and hormone actions must play specific mechanistic roles in malignant transformation and cancer progression. Here we will review the mechanisms of sex determination and highlight how the resultant sexual dimorphism could impact on oncogenesis and sex differences in cancer rates. Oncogenesis involves among other things, alterations in epigenetics, gene expression, metabolism, immune function, and angiogenesis. Each of these areas is a target of sex determination, and therefore we will discuss each at different major epochs of development including pre-implantation, post-implantation, puberty, and adulthood (Fig. 2).

### Pre-implantation

The pre-implantation stage of development provides a unique window on the distinct biologies of male and female sex chromosome complements and their effects on gene expression, metabolism and growth. During the period preceding X-inactivation and formation of the placenta, embryo biology is dictated by the differences in chromosome complement and direct sex chromosome effects on genome-wide epigenetics, gene expression, and metabolism. Decades of observations have demonstrated that under most conditions, male embryos grow faster than female embryos [132–134]. This is true in multiple eutherian species, including humans, as well as in birds [135]. At this earliest stage of development, differences in growth rate may be determined most potently by differences in glucose and amino acid metabolism, mitochondrial complement, and in rates of apoptosis [133, 134, 136–140]. Differences in metabolism are driven most directly by differences in gene dosage of X encoded metabolic regulators whose expression can be further modified by imprinting. Sex differences in expression of the X chromosome encoded and cancer metabolism-related genes glucose-6-phosphate dehydrogenase (G6PD) and O-Linked *N*-acetylglucosamine (GlcNAc) transferase (OGT), differ at both the mRNA and protein level in the blastocysts of multiple species and are correlated with measurable differences in glucose metabolism [141–143]. During the first week post-fertilization, the embryo shifts from using pyruvate to glucose as an energy source [133]. During this time, pyruvate uptake by early male embryos exceeds that of females, and direct measurements of total glucose metabolism and pentose phosphate shunt activity indicate that male embryos metabolize greater quantities of glucose but pentose phosphate shunt activity is four-fold greater in female embryos [136, 144]. In addition, measures of glutamine metabolism indicate that Kreb cycle activity is also greater in male embryos, possibly consistent with their greater mitochondrial DNA content [136]. Differences in metabolism are correlated with measurable differences in the speed of embryo growth and development [132, 145, 146]. The relatedness of differences in glucose metabolism to cancer growth is an enormous area of active research [147]. Whether sex differences in metabolism are evident in brain tumors and how differences in metabolism might affect the thresholds for transformation in brain tumors has yet to be addressed but will undoubtedly be an important application of the biology of sex differences in the development of metabolic approaches to cancer care.

Prior to X-inactivation sex differences in blastocyst epigenetics are evident. A component of sex-specific epigenetics is attributable to O-Linked *N*-acetylglucosamine (GlcNAc) transferase (OGT), which is more highly expressed in female embryos [151]. OGT provides a link between metabolism and histone H2B O-GlcNAcylation [152] and has recently been implicated in the aberrant epigenetics of cancer [153]. In addition, KDM6A (UTX) is an X encoded lysine demethylase and an essential regulator of histone H3 lysine K27 (H3K27) methylation [154]. The status of H3K27 trimethylation is critical for normal development and differentiated function. The importance of appropriate regulation of H327 methylation is underscored by the frequent mutation of H3K27 to methionine in pediatric glioblastoma [32–39]. In this regard, H3K27 demethylase activity may differ in male and female cells. UTX escapes X inactivation [155] and its Y chromosome paralog UTY does not possess the same degree of catalytic activity [156]. UTX has already been suggested to play a sex-specific tumor suppressor role in T cell acute lymphoblastic leukemia [157].

The most singular difference between male and female epigenetics at this stage is the random inactivation of either the maternally or paternally acquired X chromosome [158, 159]. This uniquely female event occurs in stages in progenitor cells and is perpetuated in all subsequent daughter cells. It results in the transcriptional silencing of most alleles on one of the X chromosomes and involves the coating of the inactivated regions by the non-coding RNA *Xist*, deacetylation of associated histones, and methylation of X chromosome DNA [160]. The extent of X inactivation is normally variable and consequently there is incomplete silencing of X encoded alleles [161]. Among the genes that escape inactivation are those with paralogs in the pseudo autosomal region (PAR) of the Y chromosome. This component of X inactivation preserves equal gene dosage in males and females, except in cases like UTX where the Y paralog does not possess equivalent activity. Escape from inactivation is not limited to PAR paralogs. Consequently, mosaicism occurs within female tissues on the basis of random X inactivation and variable X gene dosage resulting in expressed heterozygosity for some maternal and paternal alleles. This is in contrast to maternal-only X chromosome gene expression in male tissues. How variable X chromosome inactivation and X mosaicism relates to sex differences in health and disease including cancer predisposition is not known. The field of induced pluripotent stem cells has revealed significant relationships between X reactivation, pluripotency, and viability [162]. This is relevant to cancer, as malignant transformation of differentiated cells requires the re-acquisition of a pluripotent-like state in the subset of cells with tumor-initiating capacity. Thus, transformation may require fundamentally different processes for male and female cells. The possibility is supported by the oncogenic effect of blocking *Xist* expression in mice [163].

### Post-implantation

Post-implantation in utero growth and development is determined by maternal conditions, placental function, post X-inactivation differences in chromosome complement, and testosterone action. The placenta is derived from fetal tissues and is thus XX or XY. It is the primary regulator of in utero homeostasis for the fetus, and placental responses to maternal stress reveal sex differences in epigenetics, vascular, immune, and hormonal function. In male placentae, microvessel dilatation in the face of maternal hypertension preserves placental perfusion, size, and fetal growth rate [166, 167]. In response to maternal nutrient deprivation, IGF2 synthesis and secretion become elevated in male placentae and male fetal growth is again preserved compared to female fetal growth [168]. The absence of these adaptations in female placentae is associated with reduction and retardations in in utero growth but increased survival. Notable among the female placental adaptations to in utero stress is the upregulation of immune system genes [169]. In response to maternal asthma, multiple cytokine genes are up-regulated in female placentae. No comparable response is observed in male placentae and evidence for higher rates of intrauterine infection in preterm male deliveries are observed [170]. OGT activity (see above) has recently been identified as one of the mediators of sex-specific placental epigenetic responses to in utero stress [171].

Sex-specific placental and fetal epigenetic responses to in utero stressors may be widespread [164]. In a fascinating study of pregnant Gambian women, micronutrient supplementation resulted in different post-natal effects on the male and female methylome [148]. These differences involved non-overlapping autosomal loci with enrichment for different functional categories. In females, methylation changes involved components of immune and non-immune anti-infective action. In contrast, none of these genes were affected by supplementation in males. Instead, target genes in males were involved in growth and development. Several imprinted genes were also differentially affected by micronutrient supplementation. Notable among these was *GNAS*, the stimulatory alpha subunit of heterotrimeric G proteins and a critical regulator of cAMP levels. We, and others, have shown that cAMP levels regulate normal and neoplastic growth in the nervous system [41, 75–78]. Moreover, we have recently shown that polymorphisms in cAMP regulators are associated with sex-specific effects on brain tumor risk in individuals with Neurofibromatosis type 1 [41]. Multiple other examples of sex-specific epigenetic responses to in utero conditions have been published, including the effect of folate on methylation in genes involved in gut development [149], and the effect of placental insufficiency on methylation of the *HSD2* gene [150].

Together these observations raise the possibility that sex differences in epigenetics allow males and females to preserve different aspects of homeostasis. In this regard, it is interesting to note that male adaptations to multiple forms of in utero stress preserve their growth rate at the expense of survival [165]. The female adaptation results in a reduction in growth rate and upregulation of immune response genes. It is interesting to consider how sex differences in baseline and stress-induced epigenetics might relate to sex differences in thresholds for malignant transformation. The oncogenic effects of abnormal histone modification and DNA methylation, together with the anti-tumor effects of histone deacetylase and DNA methyltransferase inhibitors, highlight the critical role that epigenetic regulation plays in cancer and therapeutic response. If male epigenomes are poised to preserve growth over function, while female epigenomes are poised to preserve function over growth, are males more susceptible to an uncontrolled growth response to genotoxic stressors than females?

After the formation of the testes, in utero sex differences are increased through testosterone action. Even transient exposure of fetal tissues to testosterone was shown more than 50 years ago to have permanent effects on phenotype and behavior [172]. These observations inspired the concept of organizational effects of sex hormones as distinct from their acute actions. In the brain, organizational effects of testosterone, primarily acting as estradiol through aromatase-dependent conversion, result in sex differences in astrocyte and neuron numbers, the size of the resident microglial population, as well as synaptic density and function [173]. Other important macroscopic sex differences in brain development include the kinetics of brain growth and myelination, and ultimate brain size and degree of myelination [174, 175]. The precise mechanisms that contribute to the organizational effects of sex hormones on brain size and function remain to be fully elucidated. They involve changes at the molecular level involving histone modification and DNA methylation, as well as at the level of differences in cellular composition and tissue structure. Sex differences in the kinetics and magnitude of microglial accumulation have been hypothesized to play a role in the differing stage of life vulnerabilities of males and females to mental illness and other neurological disease that exhibit significant sex differences [102]. As discussed above, microglia accumulate in brain tumors and have been shown to exert both pro- and anti-tumor effects. Whether sex differences in microglial accumulation are evident in brain tumors and what, if any, effect sex differences in microglia numbers and function may have on brain tumor incidence and outcome has yet to be directly addressed.

Using a model of glioblastoma, we explored the consequences of in utero sex determination on malignant transformation. We found that in response to progressive perturbation of growth regulation through step-wise loss of neurofibromin and p53 function and activation of the EGF receptor pathway, male murine astrocytes exhibited accelerated growth, expansion of a clonogenic stem-like cell fraction, in vitro soft colony formation, and in vivo tumorigenesis [14]. Female astrocytes did not exhibit similar growth and neoplastic changes. Resistance and susceptibility to transformation were related to differences in Rb regulation and again suggest that males and females are poised to respond to perturbations that threaten normal growth in fundamentally different ways that may have great relevance to cancer risk and response to therapeutics. The relative contributions of sex chromosome complement, and the organizational effects of sex hormones to sexual dimorphism in Rb regulation, remain to be measured.

### Puberty and beyond

Among the profound consequences of in utero sex determination are the organization of tissues to respond to signals at the onset of puberty, which manifests in functional gonads, additional organizational and acute activational effects of sex hormones, and secondary sex characteristics. Post-puberty differences in estrogen and testosterone levels exert potent control over tissue structure and function and organismal behavior in health and disease [16]. The impact of sex hormones on human disease is indicated by numerous correlates between disease incidence and changing levels of sex hormones, as well as in experimental settings when hormone action is manipulated by gonadectomy or treatment with exogenous steroids. A series of studies have examined estrogen effects in experimental models of medulloblastoma. Primary patient tissues and cell lines, as well as genetically engineered mouse models of medulloblastoma demonstrate expression of estrogen receptor β isoforms [176, 177]. Estrogen receptor activation promotes medulloblastoma growth in experimental models [178] suggesting a potential benefit for estrogen receptor blockade in medulloblastoma treatment [176].

### Pregnancy and cancer

Cancer incidence increases slightly following pregnancy, and higher rates of incidence are found in women who delay first pregnancy to late in life, suggesting hormone-mediated effects on cancer risk [179]. However, acute hormone actions may not be the only way in which pregnancy affects cancer incidence and progression. Recently, the potential for fetal microchimerism, in which fetal cells persist post-partum in maternal tissues, to play a unique role in women’s health and disease has been described [180]. Fetal microchimerism appears to have several protective and potentially deleterious effects including promotion of wound healing but instigation of autoimmune disease. Several provocative findings suggest that fetal microchimerism may play either a protective or a pro-tumorigenic role in some women’s cancers [181–185]. How fetal microchimerism might affect brain tumor biology and outcome has yet to be addressed.

## Evolutionary basis for sex differences

### Evolutionary mechanisms related to sex differences in growth and cancer

**Fig. 3:**
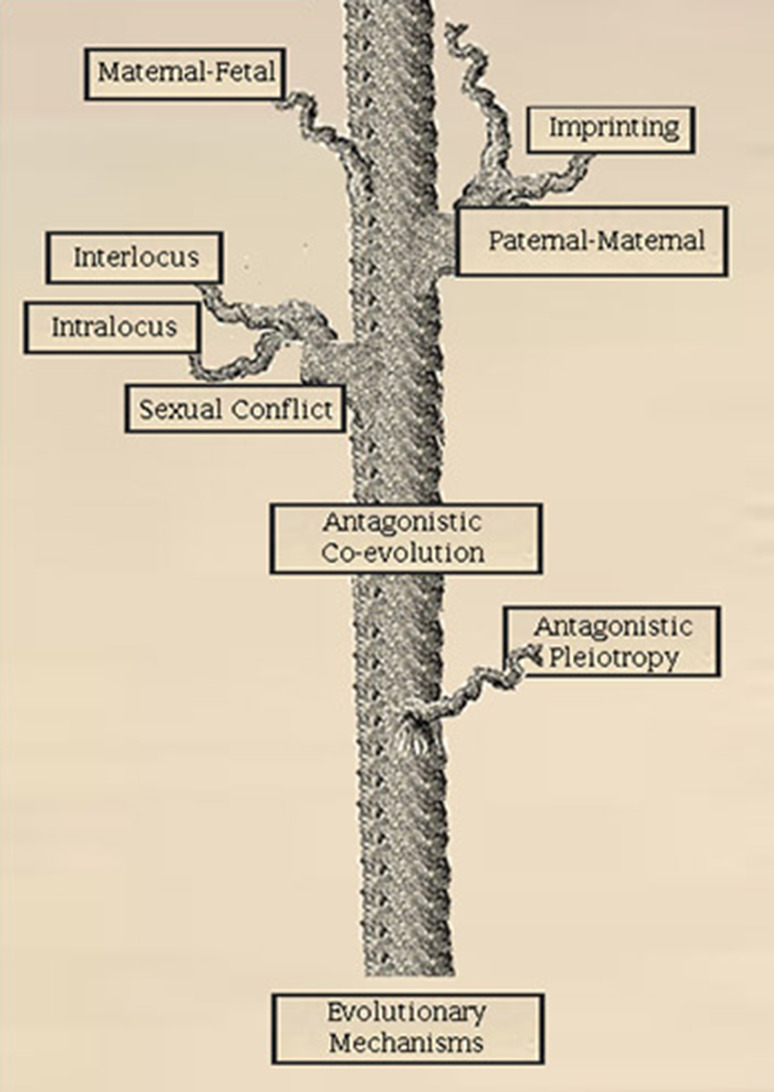
Evolutionary processes underlying sex differences. A framework for organizing evolutionary processes that can impact on cancer risk with an emphasis on those that could underlie sex differences in cancer risk. The trunk and branches illustrate mechanistic relationships. No phylogenetic relationships are inferred

Many sex differences that play a role in cancer risk may be by-products of evolutionary processes. Natural selection does not result in fully optimized traits for all individuals at all times. Instead, the relative fitness, or adaptive advantage, a trait confers on the organism is always a compromise or trade-off between competing fitness interests. There are two general classes of such trade-offs: antagonistic pleiotropy and antagonistic coevolution. Antagonistic pleiotropy occurs when a genetic variant confers a fitness advantage for an organism in one respect (perhaps with respect to reproductive success), but may be disadvantageous in another respect (perhaps by raising cancer risk and thus reducing life span); CAG repeats in the androgen receptor in men may be a vivid case of this, discussed below. Antagonistic coevolution is genetically based conflict between entities with competing fitness interests; such conflicts can occur between hosts and parasites, mothers and fetuses, or, males and females, in which case it is called “sexual conflict.” Sexual conflict may lead to the evolution of divergent genetic and epigenetic systems involved in immune response, metabolism, growth, and aging. The genes involved in such systems may promote differential cancer risk as a secondary effect [186]. Here we will review evolutionary mechanisms that may contribute to sex differences in cancer risk. In particular, we hypothesize that intralocus sexual conflict (displacement of the sexes from phenotypic optima as a result of sex-specific selection on shared traits regulated by shared genetic machinery) may play a significant role in differential cancer risk. Sex differences in growth regulation may establish different thresholds for malignant transformation in males and females and thus account for the differences in their cancer rates (Fig. 3). Understanding the selected mechanisms governing such thresholds will be essential, in our view, to more effective cancer care.

### Antagonistic pleiotropy

The antagonistic pleiotropy model of cancer risk suggests that alleles with late-acting deleterious effects are maintained in the population by active selection because these same alleles have benefits during early developmental and/or reproductive stages. Thus, for instance, increased height or weight in males might increase one’s chances of competing successfully for a mate, but might also in turn raise one’s risk of cancer later in life. Large size is associated with greater cancer risk, particularly in fast-growing tissues such as bone and brain. Recent evidence suggests this may be related to the strong correlation between lifetime risk of cancer and the number of divisions within self-renewing cell populations [187]. Interestingly, pediatric cancers most commonly involve bone marrow, bone, and brain [186], and are significantly more common in males than females [15, 188]. Thus, large size and rapid development in males may increase their fitness but also increase their cancer risk [189]. Summers and Crespi argue that the CAG region of the androgen receptor is a locus of antagonistic pleiotropy. Short repeats are associated with increased transactivation of the androgen receptor and increased fertility, but also increased risk of prostate cancer and more aggressive forms of disease [190]. In contrast, increased CAG repeat length is associated with hypo-androgenicity, low rates of spermatogenesis, and reduced male-specific traits.

There does not appear to be similar selective pressure for large size in females. In fact, there are multiple lines of evidence to indicate that growth optima in males and females are quite different. Consider paternal and maternal imprinting effects on the pro-growth gene IGF2 and the growth suppressive gene H19. Paternal imprinting of H19 blocks expression of the paternally derived allele, while maternal imprinting of IGF2 blocks expression of the maternally derived allele. This may be a case where selective pressures to protect the differential growth optima create different vulnerabilities to genetic events that alter growth regulation. Males may be more vulnerable to loss of tumor suppressor or gain of oncogene function because this moves their growth regulation towards their growth optima. In contrast, females may be more resistant to the same cancer-initiating events, because such events displace them from their optima, and trigger counter regulatory homeostatic mechanisms. Hypotheses like these can be experimentally addressed in model systems like the one we have used to explore sex differences in thresholds for transformation upon loss of p53 and Rb function [14]. This may be a vivid example of intralocus sexual conflict (described above), mediated by imprinting, though likely also affected by differential regulatory pathways of gene expression in males and females.

Many genes associated with cancer also play important functional roles in other contexts, such as in angiogenesis, placentation, spermatogenesis, and control of tissue differentiation and development. Thus, the maintenance of these genes may be simply due to other positive effects at other stages in life history. It is possible that the differential prevalence, mortality, and progression of cancer in males versus females are due to antagonistic pleiotropy, i.e., traits selected to enhance male or female fitness (higher growth rates and larger size, or early menses) that coincidentally increase cancer risk later in life, or, antagonistic coevolution, such as the case of differential regulation of growth optima, described above.

### Sexual selection

Sexual conflict is expected because divergent reproductive strategies of the sexes lead to different selection pressures on shared traits. Sexual conflict has a variety of potential impacts, such as the evolution of displays that reduce survival. This section will focus on how sexual selection could drive cancer risk. Sex-specific optimization of reproductive strategies could result in different life spans and aging rates, and could cause sexually antagonistic selection on shared genetic architecture.

Why are sex-specific optima expected to be different? According to Adler et al. [191], the sexes have different “opportunity landscapes,” where the sex that competes more strongly for access to mates tends to pursue a “live fast, die young” strategy, sacrificing longevity for reproductive opportunity. Thus, males are predicted to invest more in traits that increase reproductive opportunity, which may in turn sacrifice longevity. Female reproduction is generally resource limited, so females compete for resources, not males.

Not all sexual conflict is due to competition for mates, however. Sexual conflict can occur any time that there are divergent optima for the sexes, either because of different basic biology (e.g., haploidy, maternal-only inheritance of mitochondrial DNA), physiology, or reproductive machinery. Of course, sex antagonism can result from natural as well as sexual selection. For example, males and females might be selected to have different body sizes for ecological reasons.

Crespi and Summers have argued that similar antagonistic coevolution may be driving selection on genes associated with cancer in human populations [186]. Male–female conflict may generate evolutionary disequilibrium in hormonal regulation, especially involving testosterone and estrogen in their effects on reproductive tissues such as prostate, testis, ovary, and breast [192–195] while intrasexual conflict, such as male–male competition, could lead to selection of growth factor genes such as IGF1 and IGF2 [196] that fuel the growth of tumors [197]. Further, conflicts of genomic imprinting, between paternal and maternal genes, often involve enhancement or suppression of cell growth [186, 198, 199].

There are a variety of indirect sources of evidence in support of intra- and interlocus sexual conflict in humans. Several cancer genes, involving regulation of hormones, sexual development, fitness, growth, and development, have been shown to be subject to positive selection [194, 195].

Intralocus sexual conflict is expected in species with intense sexual selection on one particular sex. Species with divergent reproductive strategies are likely to exhibit highly sex-specific patterns of selection on longevity and aging. In species with intense male–male competition, male fitness may depend on large body size, and males may be selected for high investment in survival until critical body size is obtained. It is likely that in the evolutionary past, selection optimized different growth optima in males and females. These differential growth optima and homeostatic mechanisms to preserve these optima may play a significant role in differential cancer risk. Understanding these mechanisms, and their evolutionary role, may be of great value in sex-specific targeted interventions on cancer—whether in the service of screening and prevention, or treatment.

## Integrating the biology of sex differences into lab-based and clinical investigations

Accounting for the complexities of sex effects on human health and disease requires an integrative approach. In the laboratory, molecular mechanisms may need to be evaluated in both male and female cell models before their true relevance to human disease can be appreciated, and preclinical studies should be conducted in both male and female animals before moving on to clinical trials. The increased size and cost of experiments and the lack of familiarity with helpful model systems, experimental paradigms, necessary controls, and potential pitfalls, all conspire to make the prospect of exploring sex differences unattractive. Here we will review several laboratory and clinical investigations that highlight important experimental paradigms in sex effect research. Without routine assessment of whether sex matters it will not be possible to completely understand brain tumors and other diseases and to fully realize the value of personalized approaches to medical care.

### Laboratory investigations

Glioblastoma research provides a vivid illustration of how important observations regarding sex differences in disease can be overlooked in laboratory and clinical investigations. It has been consistently noted that more males are affected by glioblastoma than females in clinical studies since the earliest publications of treatment responses [200]. Moreover, shorter event-free survival in males has been repeatedly noted [188]. Despite this common knowledge about these key differences between males and females, there has been no coordinated effort in the clinical arena to prospectively determine whether treatment approaches should be modified for males and females. In the laboratory setting, there have been a small number of relevant and important studies. Reilly and colleagues, using a mouse model of astrocytoma involving concurrent germline heterozygous loss of neurofibromin (Nf1) and p53 tumor suppressor function, demonstrated a male prevalence in glioma formation [201–203]. The sex disparity in gliomagenesis was strain specific and Bl/6 mice appeared to be more susceptible to tumor formation than the 129S4/SvJae line. In addition, the sex difference in glioma rates was dependent on the parent origin of the genetic mutations. We also found that combined loss of Nf1 and p53 function in differentiated astrocytes had a male predominant effect on transformation and tumorigenicity [14]. However, when combined loss of Nf1 and p53 was engineered in neural stem cells, a high rate of tumor formation was observed without sex differences [204]. Together these observations suggest that the magnitude of sex effects may vary as a function of strain and tumor cell of origin/lineage effects.

In our model of *Mesenchymal* GBM, the effect of sex on transformation was evident in the absence of sex hormones, and thus independent of their acute activational effects. In vivo tumorigenesis was also unaffected by whether male or female implants were made into male or female mice. In contrast, in a U87 xenograft model, female recipient nude rats survived longer than their male counterparts [205]. Estrogen receptor beta activation has been shown to inhibit the growth of human GBM cells [206]. These findings may indicate that there is a tumor suppressive function of estrogen.

Ruling out acute activational effects of sex hormones on transformation and tumorigenesis in our model means that these sex differences must arise either as a result of the differences in chromosome complement or the organizational effects of sex hormones. The four core genotypes mouse model can distinguish between prenatal effects of sex chromosome complement and sex hormones [207]. In these mice, the testes determining gene Sry has been transferred from the Y chromosome to an autosome on a Bl6 background. Thus, four genotypes are possible: XX^Sry−^, XX^Sry+^, XY^Sry−^, XY^Sry+^, resulting in XX/gonadal females, XX/gonadal males, XY/gondal females, XY/gonadal males, respectively. Post-natal gonadectomy with or without hormone replacement/substitution can delineate additional organizational effects as well as acute activational effects of sex hormones [208]. Additional genetic models are available to manipulate expression of X alleles that escape X inactivation [209, 210] or Y chromosome gene expression with consomic Y strains [211].

### Sex differences in genomic studies

Detecting sex differences in polymorphic risks, mutational frequencies, or epigenetic landscapes requires analyzing male and female specimens separately. We recently demonstrated the value of this approach in identifying sex-specific modifiers of glioma risk in individuals with neurofibromatosis type 1 [41]. Our studies confirmed that the cAMP pathway was an important modifier of gliomagenesis in humans and revealed for the first time that cAMP may have a sex-specific effect on glioma. Minor allele variants in adenylate cyclase 8 (AC8) increased the risk for glioma in female patients but decreased risk in male patients. These data indicate that important differences in oncogenic mechanisms between males and females may be overlooked when specimens are analyzed together.

Sex-specific effects of polymorphisms are not limited to AC8. A single nucleotide polymorphism (SNP) in the MDM2 promoter (SNP309: rs2279744) also exhibits sex-specific effects on cancer rates [212]. MDM2 is a negative regulator of p53 function. It is frequently amplified in human cancers resulting in loss of p53 function. Two alleles are described for SNP 309: the GG and the GT genotypes. The GG allele is associated with increased MDM2 protein levels and reduced p53 function [212]. The GG allele has been associated with early disease onset in various cancers. For instance, the SNP309 has been shown to be associated with accelerated formation of melanoma and colon cancer only in women, [213, 214] and even associated with the early onset of pediatric acute lymphoblastic leukemia (ALL) in girls (Median age of diagnosis: girls 36 months versus boys 60 months). [212]. The GG genotype of the MDM2 promoter may increase the binding affinity of the transcription factor Sp1. Interestingly, Sp1 is a well-characterized co-transactivator of steroid receptors such as estrogen receptor and estrogen receptor may thereby modify the influence of SNP309 on MDM2 function in a sex-dependent fashion [214]. How sexual dimorphism at the level of MDM2 transcription might interact with sexual dimorphism at other levels of the p53 pathway remains to be determined.

## Future directions

Ultimately the elucidation of sex differences in cancer incidence and therapeutic response will mandate adaptation of current cancer screening and treatment approaches for sex-specific application. Laboratory-based investigations will need to consider sex differences from the cellular to the organismal level and preclinical studies should be done in male and female animal models. Cancer screening programs will need to stratify risk based on sex-specific effects of polymorphisms and mutations. Prevention programs will need to consider sex differences in the molecular mechanisms of cancer as well as social and behavioral influences on exposures like tobacco, the sun, or industrial chemicals. Finally, treatment may need to be tailored to male and female patients based on disparate molecular oncogenic mechanisms, different vulnerabilities to specific toxicities, as well as differences in how their epigenomes are poised to respond to the genotoxic, physical, psychological, and social stresses of cancer treatment.
